# Indole-3-acetic-acid-induced phenotypic plasticity in *Desmodesmus* algae

**DOI:** 10.1038/s41598-018-28627-z

**Published:** 2018-07-06

**Authors:** Tan-Ya Chung, Chih-Yen Kuo, Wei-Jiun Lin, Wei-Lung Wang, Jui-Yu Chou

**Affiliations:** Department of Biology, National Changhua University of Education, Changhua, 500 Taiwan

## Abstract

Phenotypic plasticity is the ability of a single genotype of an organism to exhibit variable phenotypes in response to fluctuating environments. It plays a crucial role in their evolutionary success. In natural environments, the importance of interactions between microalgae and other microorganisms is generally well appreciated, but the effects of these interactions on algal phenotypic plasticity has not been investigated. In this study, it revealed that indole-3-acetic acid (IAA), the most common naturally occurring plant hormone, can exert stimulatory at low concentrations and inhibitory effects at high concentrations on the growth of the green alga *Desmodesmus*. The morphological characteristics of *Desmodesmus* changed drastically under exposure to IAA compared with the algae in the control environment. The proportion of *Desmodesmus* unicells in monocultures increased with the IAA concentration, and these unicells exhibited less possibility of sedimentation than large cells. Furthermore, we discovered that lipid droplets accumulated in algal cells grown at a high IAA concentration. Results also demonstrated that the presence of algal competitor further stimulated inducible morphological changes in *Desmodesmus* populations. The relative abundance of competitors influenced the proportion of induced morphological changes. The results indicate that phenotypic plasticity in microalgae can be a response to fluctuating environments, in which algae optimize the cost–benefit ratio.

## Introduction

Phenotypic plasticity is the capacity of a single genotype of an organism to alter its phenotypes in response to different environments^1^. Most organisms exhibit phenotypic plasticity, which enables them to adapt to fluctuating selective pressures. The ability to overcome environmental challenges is critical for the evolutionary success of organisms. Stress-induced defense is a typical example of phenotypic plasticity that has evolved from single-celled organisms to vertebrates and plants^2,3^. Franssen *et al*. analyzed the morphological flexibility in fish living in both stream and reservoir habitats^4^. They revealed that the body shape was drastically and consistently different in the fish in reservoir habitats compared with the populations in streams. Furthermore, the fish analyzed in reservoirs had shallower bodies with smaller heads compared with the fish from streams. Fraser *et al*. also reported that the abaxial stomatal density and leaf area of bluebunch wheatgrass (*Pseudoroegneria spicata*) was plastic in response to water and temperature manipulations^5^. Corno and Jurgens studied the effect of grazing and substrate supply on the population size structure of a bacterial strain (*Flectobacillus* sp.), which exhibited noticeable morphological plasticity^6^. When the predator bacterivorous flagellate *Ochromonas* sp. appeared, the bacteria developed filamentous forms or chains of cells and appeared to be resistant to predation. In aquatic ecology and evolution, Cladoceran species (*Daphnia* spp. and *Moina* spp.) are important model organisms and are textbook examples of inducible defense against predators. *M*. *macrocopa* exhibited earlier reproduction time and increase in offspring number when exposed to fish (*Rhodeus ocellatus*) kairomone^7^. In the presence of midge larva (*Chaoborus americanus*), *D*. *pulex* produce a socalled neck spine which may carry several teeth. Furthermore, females exposed to midge larva often delay their maturation^8^. These morphological and physiological modifications are supposed to serve as an anti-predator device^9^. Interestingly, Yang *et al*. shown that protozoan flagellate *Ochromonas* sp. grazing could induce colony formation in the cyanobacterium *Microcystis aeruginosa*, whereas *M*. *aeruginosa* populations in the control and the grazing treatments of copepod *Eudiaptomus graciloides*, cladoceran *D*. *magna*, and rotifer *Brachionus calyciflorus* were still strongly dominated by unicells and paired cells and no colony forma occurred^10^. Generally, mobile organisms often migrate from unfavorable environments. Thus, phenotypic plasticity has been suggested to be more critical for immobile organisms than mobile organisms.

A coenobium is a type of colony that is formed in algae. It has a definite number of cells arranged in a specific manner, but these cells exhibit little or no specialization and are definitely organized. It occurs in some clades of algae such as *Desmodesmus*, *Pediastrum*, and *Scenedesmus*, which are typical planktonic algae in freshwater communities. The number of cells in a colony occurs in multiples of two, and *Desmodesmus* and *Scenedesmus* are usually composed of 1, 2, or 4 cells arranged in a single row in an oblong-ellipsoid shape^11,12^. The coenobia of *Pediastrum* are flat and one cell thick, and the uninucleate cells are arranged in concentric circles, with the marginal cells exhibiting horn-like shapes^13^. The coenobia are developed by divisions of a single mother cell, but the daughter cells stay connected through a common cell wall. Therefore, a coenobium is a typically formed colony; a clonal colony is created by the descendants of single mother cells, and the cells are often embedded in a mucilaginous matrix^14^.

The microalgae *Scenedesmus incrassatulus* is often found in freshwater ecosystems, water polluted with heavy metals, and wastewater facilities. Research revealed that divalent cation metals induce its unicellular forms, and hexavalent chromium produces misshapen coenobia corresponding to various stages of autospore formation^12^. Phenotypic plasticity is also demonstrable in the transformation of the shape of coenobial cells of *Pediastrum duplex* var. *duplex* reared at various pH levels^15^. However, phenotypic plasticity is a response to not only abiotic factors, but also biotic factors. *Scenedesmus* is well recognized for its phenotypic plasticity in adapting to grazing pressure^16^. Upon interaction with infochemicals produced by herbivores (water fleas, *Daphnia* spp.), *Scenedesmus* populations become dominated by four- and eight-celled individuals instead of unicells. *Scenedesmus* individuals that are formed by colonies can effectively prevent herbivory. Thus, grazer-induced colony formation is an evolutionary strategy for *Scenedesmus* to temporally deter feeding by small invertebrate grazers. However, environmental variation encompasses both abiotic and biotic components of the environment, including interactions among organisms. Yang and his colleagues further address how abiotic factors affect plastic responses to biotic factors. They indicated that herbicide exposure and metal (Cu)-contaminated waters both impair antigrazer colonial morphs in *S*. *obliquus*. This phenomenon can increase the risk of predation by herbivores, thereby disrupting the inducible phytoplankton community^17,18^. In contrast, they also showed that the morphological defense in *S*. *obliquus* was enhanced with more eight-celled colonies formed in populations grown in a high-CO2 environment^19^. Thus, the predator-prey interactions between herbivores and phytoplankton can be potentially changed more seriously than previously considered.

Microalgae are microscopic unicellular organisms typically found in freshwater and marine ecosystems, and they coexist with many other microbes. In natural bodies of water, the crucial physical associations and biochemical interactions between microalgae and other microorganisms are generally well recognized, and the significance of these interactions to algal phenotypic plasticity has attracted much attention^20^. Indole-3-acetic acid (IAA) is the most common naturally occurring plant hormone of the auxin class, and it regulates many aspects of plant growth and development^21,22^. Studies have demonstrated that IAA produced by microbes could help plant seed germination and root elongation^21–24^. Through phylogenetic analyses, researchers suggested that IAA biosynthesis evolves independently in microalgae, fungi, bacteria, and plants^25^. Research proved that IAA can regulate the physiological responses and gene expression in these organisms^26^. The repeated and independent evolution of IAA suggests that natural selection might have favored IAA as a universal physiological code in these microorganisms and their interactions^25–28^. IAA can act as a signaling compound between different organisms, it can be exported by the one species and imported into either organism. Thus, IAA was proposed to be a diffusible signal and to be involved in interspecific communication among different organisms^25^.

Phenotypic plasticity in coenobial algae has been documented in response to a wide variety of conditions; however, IAA has not been comprehensively documented as phenotypic plasticity inducers. Here we proposed that the IAA acts as a diffusible signal in the communication between the microalgae and its associated microorganisms. To test this hypothesis, we investigated the induced colony changes, population growth, and physiological changes of *Desmodesmus* at different IAA concentrations. We also measured the influence of the relative abundance of algal competitors on the proportion of induced morphotypes in cultures. However, the biochemical composition of IAA-induced morphotypes may be slightly changed with their protein and the fatty acids in the cells and thereby their sinking. Therefore, the sinking rates of *Desmodesmus* populations cultured in different levels of external IAA were also determined.

## Materials and Methods

### Isolation and culture of microalgae

Native species of microalgae were isolated from natural water bodies and bark of *Alstonia scholaris* from Changhua County, Taiwan. The water samples with visible microalgal populations were centrifuged at 3000 × *g* for 10 min at room temperature to concentrate cells and were spread uniformly by using sterile 4 mm glass beads onto CA agar plates. The bark samples were transferred to sterilized microcentrifuge tubes (1.5 mL) with 200 μL of sterilized water and vortexed for 5 min at 3000 revolutions per min (rpm) using a vortex mixer (AL-VTX3000L, CAE technology Co., Ltd.). Then, 100 μL of the supernatant was spread on the CA agar plates to obtain the algae from the surfaces of the bark. To isolate the axenic colony from the samples, a streak plate method was used to isolate pure algal cultures. The algae were cultured in CA medium^29^, and then the pH was adjusted to 7.2. Algal cells were stored at −80 °C with 15–20% glycerol.

### Growth conditions for algae

The microalgae were cultured for 7 days in CA medium at 125 rpm in a tube rotator and grown at 25 °C under cool-white fluorescent light (approximately 46.30 µmol m^−2^ s^−1^) with 14 hours of light and 10 hours of darkness per day (LD 14:10). Each algal culture sample was observed for cellular growth rates by measuring the optical density at 680 nm. The regression equation between cell density (y × 10^5^/mL) and OD_685_ (x) was derived as y = 162.1x + 1.3463 (r^2^ = 99.34%)^30^.

### Algae identification

The algal cells were harvested through centrifugation at 3000 × g and 25 °C for 10 min. The genomic DNA used for analysis was isolated using the AccuPrep GMO DNA extraction kit (Bioneer, Korea). The 18S rDNA was amplified through polymerase chain reaction (PCR) with the following primers: 18S forward, “TTTCTGCCCTATCAACTTTCGATG”; and 18S reverse, “TACAAAGGGCAGGGACGTAAT”. This thus yielded a fragment of approximately 1200 bp^31^. The PCR conditions were as follows: initial denaturation at 96 °C for 4 min, 36 cycles of denaturation at 96 °C for 30 sec, annealing at 50 °C for 30 s, and extension at 72 °C for 1 min, followed by a final extension at 72 °C for 6 min. The ITS1-5.8 S-ITS2 region of rDNA was amplified using the following primers: ITS forward1, “ACCTAGAGGAAGGAGAAGTCGTAA”; and ITS reverse1, “TTCCTCCGCTTATTGATATGC”. This thus yielded a fragment of approximately 1200 bp^31^. The PCR conditions were as follows: initial denaturation at 96 °C for 4 min, 36 cycles of denaturation at 96 °C for 30 s, annealing at 48 °C for 30 s, and extension at 72 °C for 1 min, followed by a final extension at 72 °C for 6 min. DNA sequencing were done by Tri-I Biotech, Inc. The Basic Local Alignment Search Tool (BLAST) was performed to find regions of local similarity between sequences on the website of the National Center for Biotechnology Information (http://www.ncbi.nlm.nih.gov).

### Lipid droplet staining

The fluorescent dyes most often used for lipid detection are BODIPY 505/515 and Nile red. For lipid detection, these fluorescent stains offer a rapid and inexpensive method of visualizing and quantifying lipid content. However, a previous reported that these dyes do not successfully stain all microalgal species^32^. Thus, we used both dyes in this assay.

### BODIPY 505/515 staining

The lipophilic fluorescent dye boron-dipyrromethene (BODIPY 505/515) (4,4-difluro-1,3,5,7-tetramethyl-4-bora-3a,4a-diaza-s-indacene) (Invitrogen Molecular Probes, Carlsbad, CA, USA) was used to monitor lipid storage in algal cells. The stain was prepared as a stock solution of 100 mg L^−1^ in anhydrous dimethyl sulfoxide (DMSO) and stored in a dark bottle. The microalgal cultures (~1 × 10^6^ cells/mL) were suspended in CA medium and stained with a concentration of 0.067 μg mL^−1^, followed by 10 min of incubation in darkness before being analyzed^33^. Cells stained with BODIPY were imaged using a Leica DM2500 fluorescence microscope.

### Nile red staining

Nile red (9-diethylamino-5-benzo[α]phenoxazinone) (AAT Bioquest, Inc.) was prepared as a stock solution of 250 mg L^−1^ in acetone and stored in a dark bottle. The microalgal cultures (~1 × 10^6^ cells/mL) were suspended in 25% DMSO and stained at a concentration of 0.5 μg mL^−1^ Nile red (10 min of incubation at 38 °C in darkness), and imaged using a Leica DM2500 fluorescence microscope^33–35^.

### Microscopic observation

Algae were harvested after each experiment, and the different-celled populations were calculated under an optical microscope (DMRB, Leica, Germany). Different algal populations (including unicellular, two-celled, four-celled, eight-celled, and large unicelluar) were calculated, and the mean numbers of cells in different phenotypes were also calculated. Additionally, the numbers of cells per coenobium were counted by dividing the total cell number by the number of coenobia.

### Scanning electron microscope observation

Algal cells were washed several times with distilled water, dehydrated using a freeze dryer (FDU-506, Eyela, Japan), coated with gold–palladium with a sputter coater (E-1010, Hitachi, Japan), and finally observed through a scanning electron microscope (SEM, TM-3000, Hitachi, Japan).

### Induction experiment under competition with *Pectinodesmus pectinatus*

To determine whether the algal–algal selection pressure further influences the sensitivity of algal responses to IAA, we performed an experiment using two different initial density proportions of two microalgae (*D*. *komarekii*: *P*. *pectinatus* = 1:1 and 1:5) with different concentrations of IAA in CA medium. The initial algal density of *D*. *komarekii* in each culture was approximately 1.0 × 10^5^ cells·mL^−1^, and the initial algal densities of *P*. *pectinatus* were approximately 1.0 × 10^5^ and 5.0 × 10^5^ cells·mL^−1^. Each treatment was performed in triplicate.

### Sedimentation experiment

#### Sinking rate

To govern the process of algal sedimentation, we used the settling column method. Briefly, the sinking rate was calculated as the change in the vertical distribution of the algal population within a sedimentation column after a finite length of time^36^. The plastic columns were 28 cm in height and 0.6 cm in diameter. In this procedure, three replicate unicellular and colonial *D*. *komarekii* cultures (3 mL) were poured in from above and were well mixed. The columns were placed in a dark cabinet at 25 °C. The distance between the two sampling points was 12 cm. Thus, the sampling point from bottom, middle and upper layer were ~0 cm, 12 cm, and 24 cm from the bottom of the plastic columns^37^. The mean sinking velocity (ʋ_SED_, m d^−1^) was calculated from the algal concentration in the settling region near the bottom layer (C_sed_), the algal concentration remaining in suspension (C_sus_), the initial algal concentration (C_0_), the height between the sampling points (h), and the elapsed time (t) according to the following equation: ʋ_SED_ = (C_sed_ − C_sus_) C_0_^−1^ h t^−1^. The significance of differences in sinking rates was determined using Student’s t test. A *P* value of <0.05 was considered statistically significant.

### Proportion of algal populations in sedimentation experiment

Algal cultures (3 mL) from the control groups and IAA-treated groups were used for the sedimentation experiment. The algal densities were measured using a hemocytometer. Then, the cultures from the control groups and IAA-treated groups were mixed well and poured into the columns that were used in the sedimentation experiment. The columns were placed in a dark cabinet at 25 °C. The distance between any two sampling points was 12 cm. After 3 and 5 h, the solution (1 mL) was obtained from sampling points in the upper, middle, and bottom regions, and the different-celled populations were calculated using an optical microscope (Leica DMRB, Japan).

### Spectrophotometric determination of chlorophyll contents

Algal cells (1.5 mL) (O.D. = ~0.8) were centrifuged at 12,000 × g and 4 °C for 5 min and the cells were lysed through freezing (−80 °C) and thawing (room temperature). Subsequently, 0.3 g of acid-washed glass beads (0.45–0.52 mm) were added and mixed gently. The mixtures were vortexed for 30 min to disrupt cells and then centrifuged at 12,000 × g and 4 °C for 5 min, and the cells were suspended with 1 mL methanol. We carried out three freeze-thaw cycles mentioned above and then incubated the mixtures at 4 °C for 24 h in a dark environment. The methanol extract was centrifuged at 12,000 × g and 4 °C for 5 min, and the absorbance of the supernatant was detected at 652 and 665 nm using a Unico spectrophotometer to estimate chlorophyll *a* and *b*. The concentrations of chlorophyll *a* and *b* per milliliter were estimated using the following equations: chlorophyll *a* (μg∙mL^−1^) = 16.29 (A665) − 8.54 (A652), and chlorophyll *b* (μg∙mL^−1^) = 30.66 (A652) − 13.58 (A665)^38^. The data were then expressed as a chlorophyll *a*/*b* ratio.

### Statistical analysis

Data values are presented as means of three replicates ±SD. The growth rates, proportions of different-celled colonies, mean number of cells per coenobium, and chlorophyll a/b ratios were compared using a one-way analysis of variance (ANOVA) with Bonferroni-Holm post-hoc test.

## Results

### Effect of exogenous IAA on algal growth

**Figure 1:**
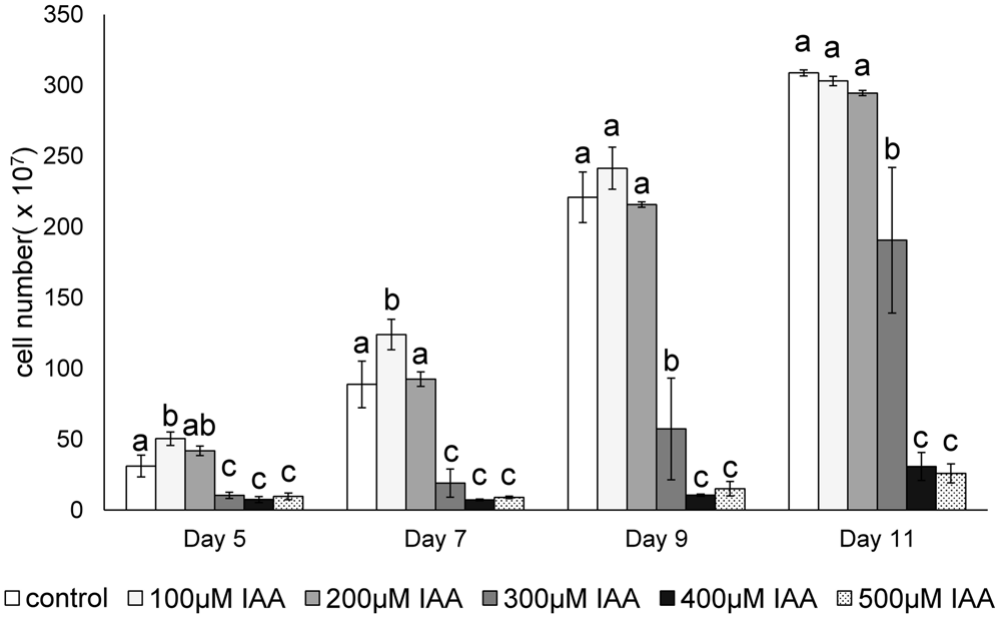
Effects of exogenous IAA on algal growth. *D. komarekii* were grown in CA medium containing different concentrations of IAA (0–500 µM). The effects of different concentrations of IAA on growth expressed as *D. komarekii* cell numbers after 5, 7, 9, and 11 days of culturing. Data are the means of three independent experiments ±SD. Means with the same letter are not significantly different from each other according to a one-way ANOVA with Bonferroni-Holm post-hoc test.

We performed a dose-response analysis to determine the fitness effects of IAA on the coenobial algae *Desmodesmus komarekii*. The results revealed that different concentrations of IAA had divergent effects on the growth of *D*. *komarekii* (Fig. 1). We found that a low concentration of IAA (100 µM) promoted the growth of algal cells after 5 days of culturing in the medium, but high concentrations of IAA (>300 µM) inhibited cell growth. Similar results were also observed after 1 week. However, a low concentration of IAA had no influence on the growth rate after day 9. By contrast, high concentrations of IAA (>300 µM) still inhibited the growth of the algal population. Thus, *Desmodesmus* can respond to the external phytohormone IAA signal and then integrate the information to initiate physiological changes. In the subsequent experiment, our aim was to determine whether the physiological cues of IAA in these cultures also trigger phenotypic plasticity responses.

### IAA-induced phenotypic plasticity in *Desmodesmus*

**Figure 2:**
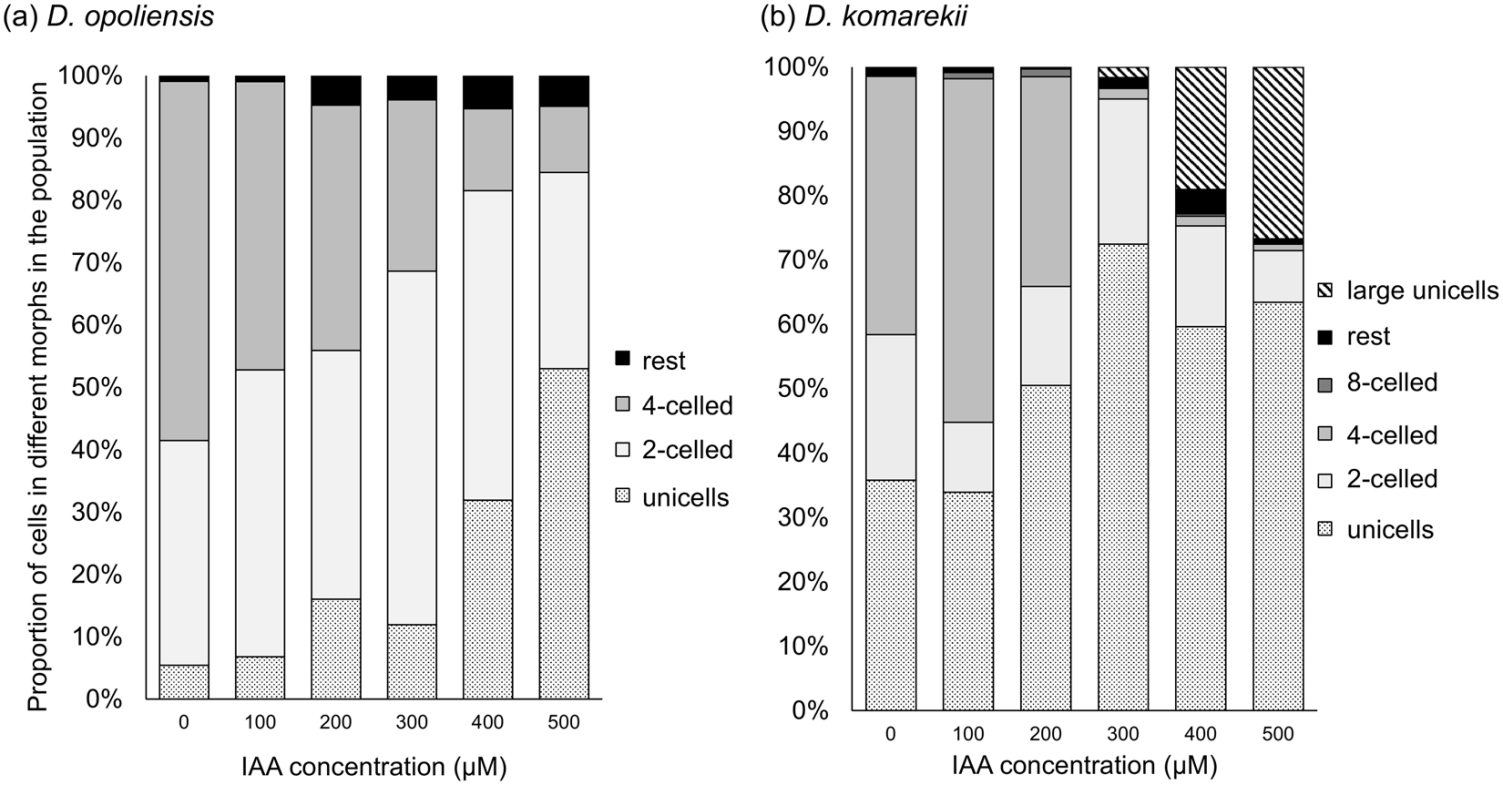
Proportions of unicells and 2-, 4-, and 8-celled coenobia in *Desmodesmus*. (**a**) *D. opoliensis* and (**b**) *D. komarekii* were cultured at different IAA concentrations. The “rest” group represents 3-, 5-, 6-, and 7-celled colonies. Data are presented as mean (n = 3) for each group, and morphotype percentages and cell types were based on 200 cell counts in each repeat.

To measure the phenotypic plasticity responses of *Desmodesmus*, we used two species. After 1 week of treatment, monocultures of *D*. *opoliensis* in the control groups (without IAA treatment) were dominated by two-celled and four-celled coenobia, with less than 10% unicells (Fig. 2a). In our experimental design, the age of the algal populations did not reach the peak of the log-phase. Thus, the initial compositions of *D*. *opoliensis* colonies are very similar to that of the cultures without IAA treatment after a week. However, the morphology of the *D*. *opoliensis* populations changed drastically at a high IAA concentration compared with that in the control environment. When the IAA concentration was increased, the proportion of four-celled coenobia declined from more than 55% to approximately 10%, and the number of unicells increased from less than 10% to approximately 50%. We observed similar results in the monocultures of *D*. *komarekii*, which were dominated by two-celled and four-celled coenobia in the control groups (Fig. 2b). When the IAA concentration was increased, the proportion of two-celled and four-celled coenobia decreased, and the number of unicells increased. The mean number of cells per particle in the control groups of these two species remained at more than 2 after 7 days of culturing.

**Figure 3:**
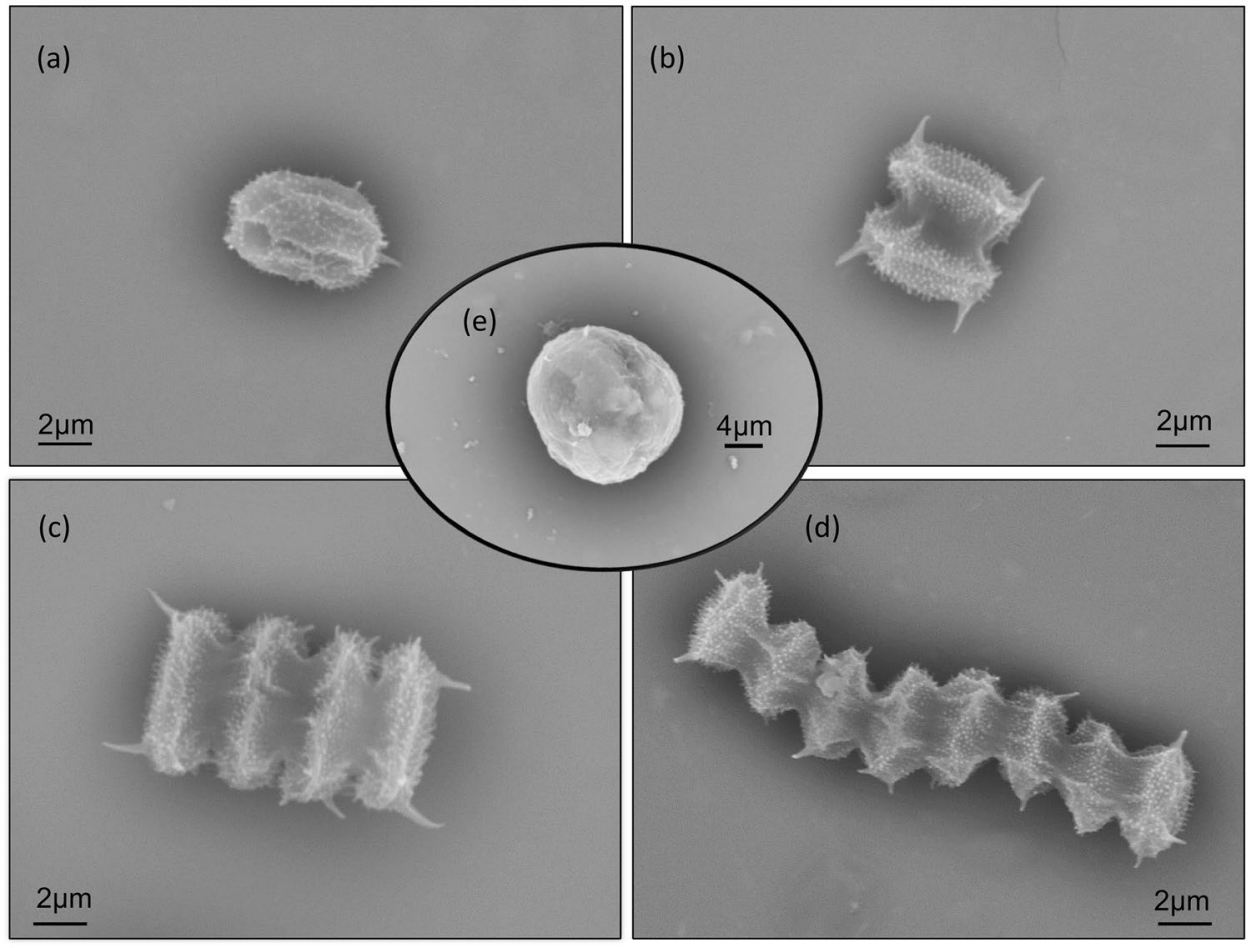
Scanning electron micrographs of *D. komarekii* cells under IAA treatment. (**a**) unicell, (**b**) two-celled, (**c**) four-celled, (**d**) eight-celled, and (**e**) large unicell colonies.

**Figure 4:**
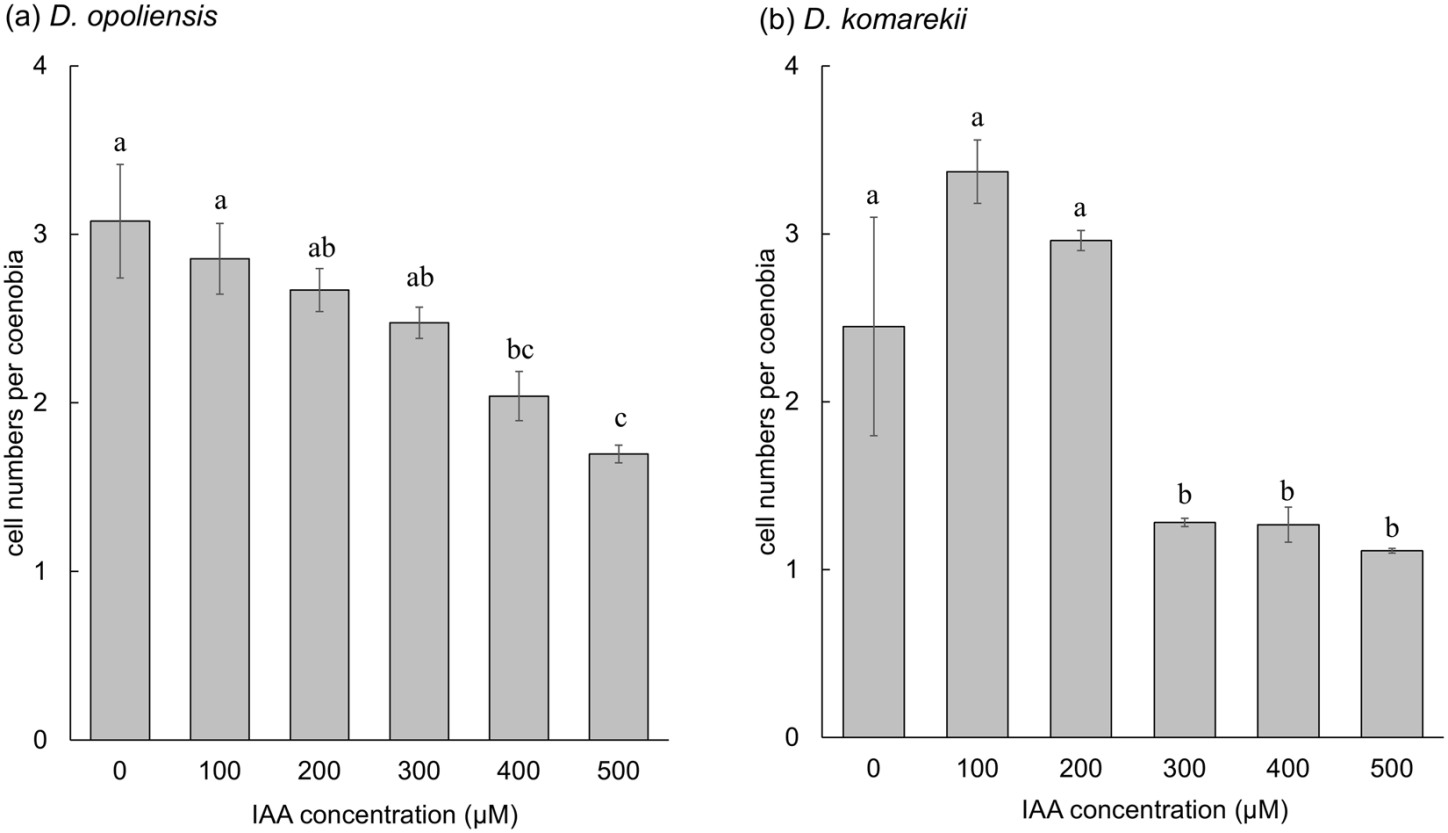
Mean number of cells per coenobium of *Desmodesmus*. (**a**) *D. opoliensis* and (**b**) *D. komarekii* were cultured in CA medium with different concentrations of IAA. Morphotype percentages and cell types were based on 200 cells counts in each repeat. Data are presented as mean ± standard error (n = 3) for each group. Means with the same letter are not significantly different from each other according to a one-way ANOVA with Bonferroni-Holm post-hoc test.

Through an SEM, we confirmed that the morphological changes in coenobia were not caused by cell aggregation but by the vegetative growth of a mother cell (Fig. 3). The connecting strands between cells were highly visible. Notably, we observed that specific large unicells were formed in the monocultures of *D*. *komarekii* under 500 µM IAA treatment (Fig. 3e). These large unicells were three times larger than the unicells. A high concentration of IAA not only affected cell growth but also induced the formation of large unicells in *D*. *komarekii*. Next, we calculated the mean number of cells per particle according to cell abundances and cells in different morphological particles. The induced colony formation of *D*. *opoliensis* decreased in the presence of IAA (Fig. 4a). The mean number of cells per particle of *D*. *opoliensis* decreased gradually as the IAA concentration gradually increased and reached its minimum level at an IAA concentration of 500 µM. Similarly, the mean number of cells per particle of *D*. *komarekii* decreased rapidly under IAA treatment, and unicells became dominant when more than 300 µM IAA was added (Fig. 4b). Growth rate inhibition signifies that microalgae are under a stressful condition. Many algal species respond to unfavorable environments by regulating their metabolism and inducing the accumulation of lipids and other compounds such as secondary metabolites and carbohydrates^39^. Thus, we subsequently investigated the differences in lipid accumulation between large unicells and other cell types of coenobia in *D*. *komarekii*.

### IAA can induce lipid accumulation in *D. komarekii*

**Figure 5:**
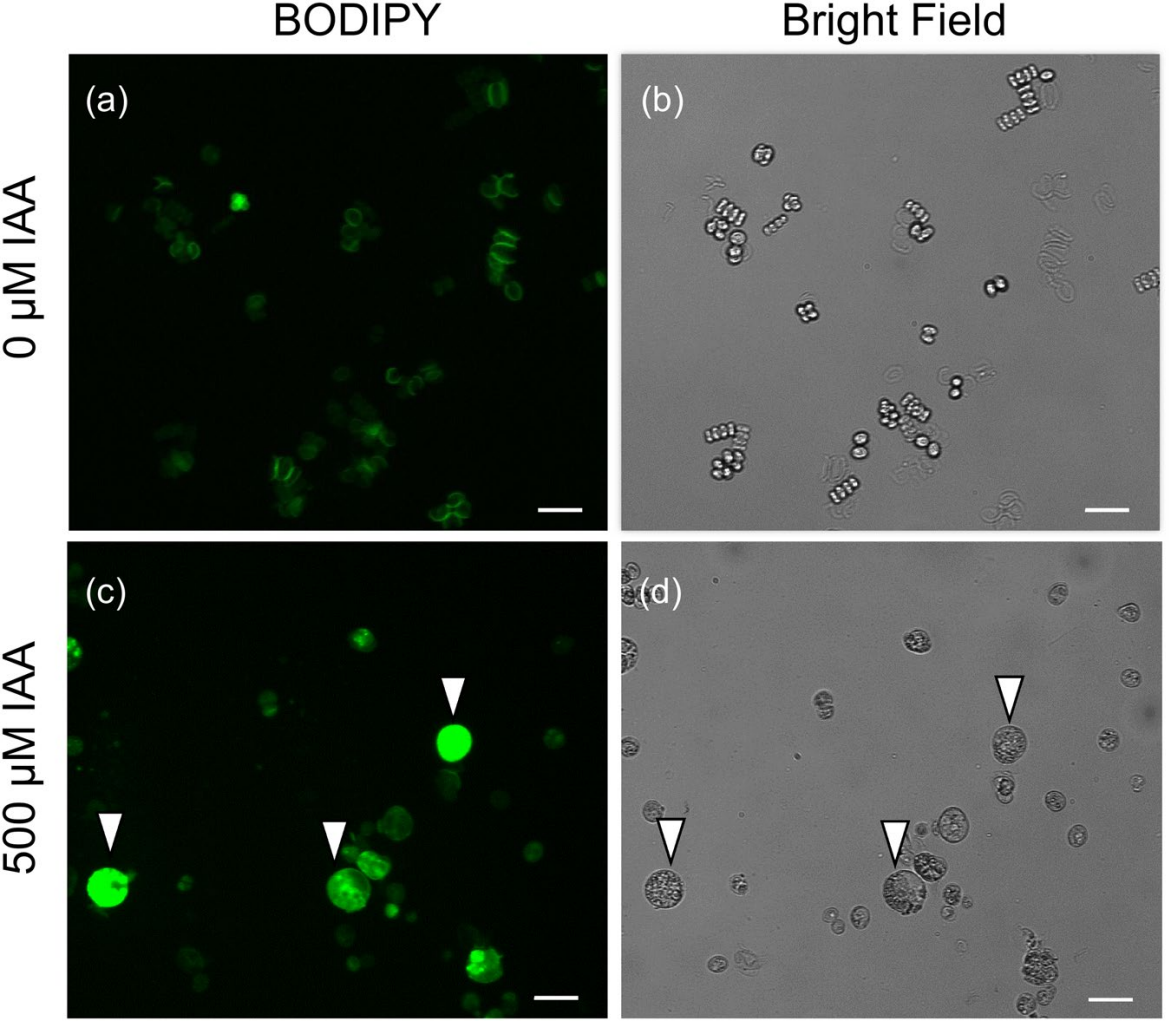
*D. komarekii* were stained with BODIPY. Arrowheads indicate the large unicell populations. (**a**,**b**) control group: (**a**) lipids visualized by BODIPY staining and (**b**) bright field. (**c**,**d**) cultured under 500 µM IAA: (**c**) lipids visualized by BODIPY staining and (**d**) bright field. Bar: 20 μm.

Living *D*. *komarekii* cells were stained with BODIPY, a reagent that yields brilliant green fluorescence in a neutral lipid environment. The results revealed that the *D*. *komarekii* cells in the control groups did not produce lipid droplets (Fig. 5a,b), but *D*. *komarekii* cells accumulated lipid after treatment with 500 µM IAA (Supplementary Fig. 1). We used Nile red dye, a reagent that yields brilliant yellow fluorescence in a neutral lipid environment, and confirmed that the *D. komarekii* cells produce lipid droplets at a high concentration of IAA. The cells stained with Nile red produced the same result as did those stained with BODIPY. The arrowheads in Fig. 5 and Supplementary Fig. 1 indicate the cells that produced lipid droplets in the algal cells. Clear qualitative differences in the level of neutral lipids accumulated within large unicells can be observed compared with the lipid amount in other cell types.

### IAA affects chlorophyll *a/b* ratio

**Figure 6:**
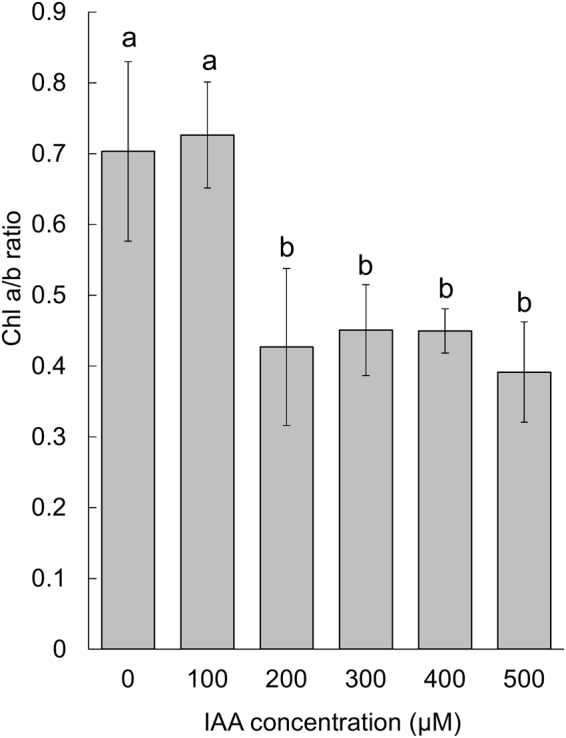
Effects of IAA treatment on chlorophyll *a/b* ratios of *D. komarekii* grown in CA medium. Mean with standard deviation. At a low (100 µM) IAA concentration and without IAA treatment, the chlorophyll *a*/*b* ratio was higher, whereas the chlorophyll *a*/*b* ratio was lower for *D*. *komarekii* grown at a high concentration (>200 µM) of IAA.

The chlorophyll *a*/*b* ratio is a measure of the proportions of light-harvesting complexes versus reaction centers, which is typically considered to be a global indicator of photosynthetic activity. As indicated in Fig. 6, at a low (100 µM) IAA concentration and without IAA treatment, the chlorophyll *a*/*b* ratio was higher; by contrast, the chlorophyll *a*/*b* ratio was significantly lower for *D*. *komarekii* grown at a high IAA concentration (>200 µM) (p < 0.05). The chlorophyll *a*/*b* ratios were approximately 0.7 (±0.13) and 0.73 (±0.07) for *D*. *komarekii* grown at IAA concentrations of 0 and 100 µM, respectively. However, the chlorophyll *a*/*b* ratios decreased to 0.43 (±0.11), 0.45 (±0.06), 0.45 (±0.03), and 0.39 (±0.07) for *D*. *komarekii* grown in media with 200, 300, 400, and 500 µM IAA, respectively.

### Colonial populations have higher sinking velocities than unicellular cells

**Figure 7:**
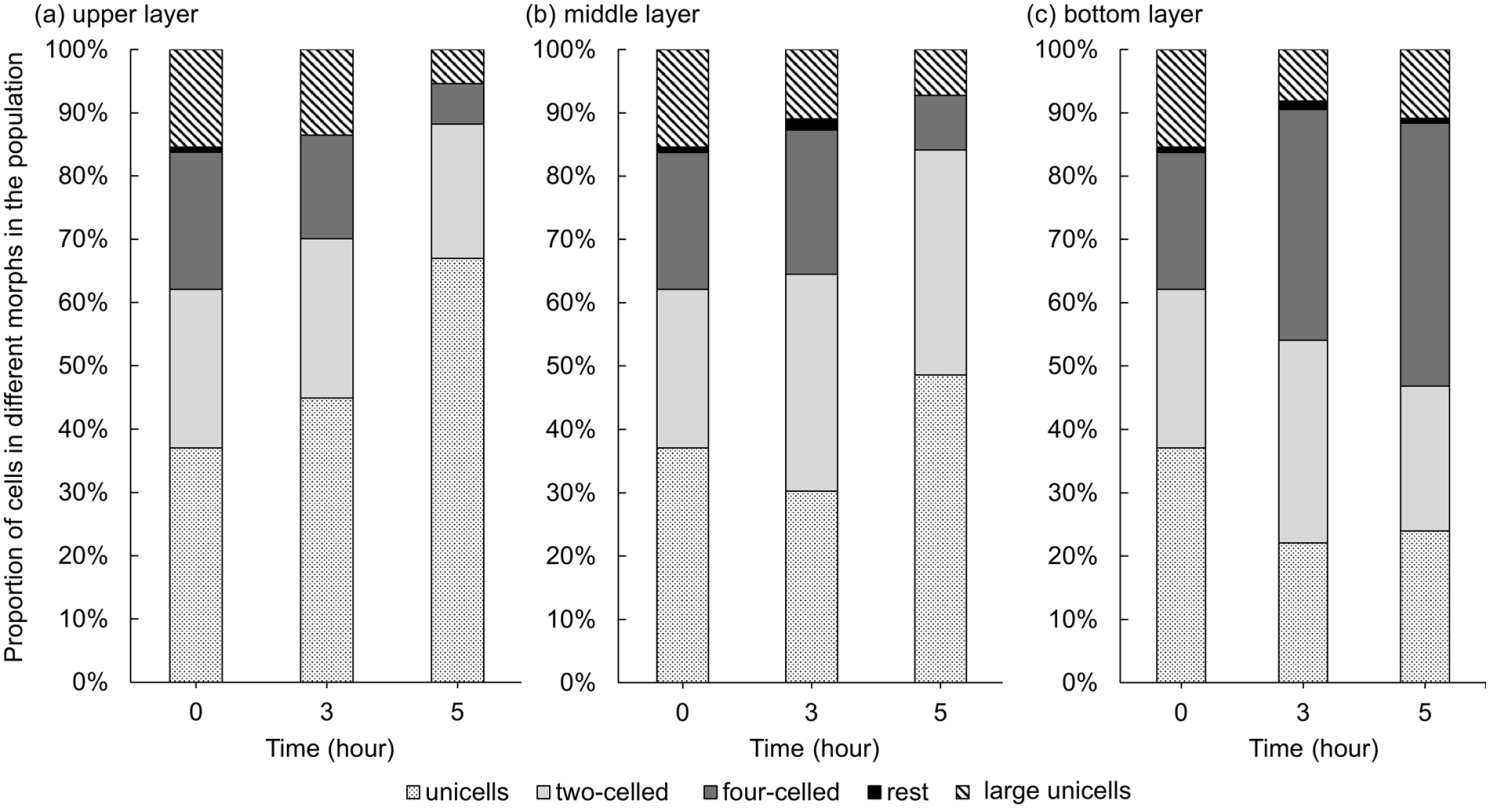
Proportions of unicells and 2-, 4-, and 8-celled coenobia in the sedimentation experiment. (**a**) Upper layer, (**b**) middle layer, and (**c**) bottom layer. The “rest” group represents 3-, 5-, 6-, 7-celled colonies. Data are presented as mean (n = 3) for each group, and morphotype percentages and cell types were based on 200 cell counts in each repeat.

**Figure 8:**
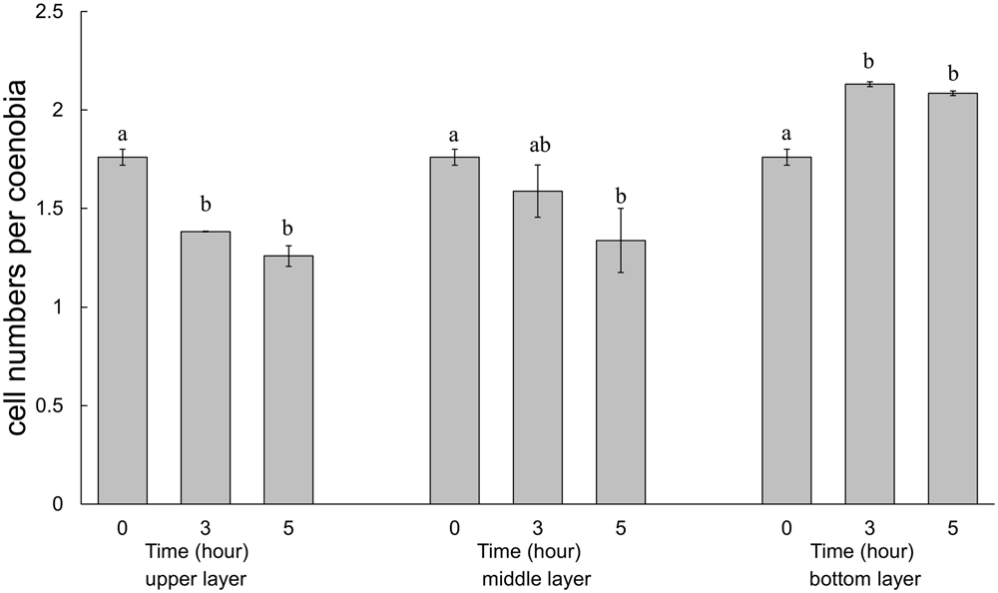
Mean number of cells per coenobium of *Desmodesmus* in the sedimentation experiment. Morphotype percentages and cell types were based on 200 cell counts in each repeat. Data are presented as mean ± standard error (n = 3) for each group. Means with the same letter are not significantly different from each other according to a one-way ANOVA with Bonferroni-Holm post-hoc test.

We revealed that most algal cells transformed into unicells at a high IAA concentration. We subsequently analyzed the difference in sinking rates among these various cell types in *Desmodesmus*. In the control groups, the proportion of two-celled and four-celled colonies accounted for the majority of colonies, and the unicells and large unicells were dominant in the cultures with 500 µM IAA (Fig. 2b). The measured settling velocities differed significantly between the control and experimental groups (under 500 µM IAA, *P* < 0.05), with the mean rates (±SD) for the control and experimental groups being 8.91 (0.061) and 1.59 (0.308) m d^−1^, respectively. Then, the cells from the control groups and the experimental groups (under 500 µM IAA) were well mixed. After 3 and 5 h, we calculated the proportions of different cell types (Fig. 7). As demonstrated shown in Fig. 7a, the proportion of unicells increased but that of four-celled colonies decreased in the upper layer. By contrast, the proportion of four-celled colonies increased but that of unicellular colonies decreased with time in the bottom layer (Fig. 7c). Notably, the large unicells were still distributed in all layers after 5 h of sedimentation. The mean number of cells per particle decreased after 3 h in the upper layer (Fig. 8a). In the middle layer, the only significant change was in the mean number of cells reduced after 5 h (Fig. 8b). In the bottom layer, the mean number of cells increased with time, and we observed significant changes in the mean number of cells after 3 h (Fig. 8c).

### Reversibility of phenotypic plasticity

We transferred the *D*. *komarekii* populations exposed to the high concentration of IAA to fresh CA media, and they gave rise to typical unicellular, two-celled, and four-celled dominant populations (Supplementary Figs 2 and 3) that had the same growth rate as those in the control (IAA-free) environment (data not shown). To further confirm the reversibility of phenotypic plasticity of large unicells, we transferred 100 μL populations exposed to a high concentration of IAA onto fresh CA plates and spread them evenly using sterile glass beads along with a gentle shaking motion. We measured large unicells by observing the plates under a dissecting microscope (MB-630, MAJOR, Taiwan). After 1 week of culturing, we used a sterile toothpick to transfer a tiny amount (barely visible to the eye) of the large unicells from the colonies and spread them onto glass slides. We then observed the population composition using an optical microscope (Leica DMRB, Germany). The results showed that the large unicells also gave rise to typical unicellular, two-celled, and four-celled dominant populations (data not shown). Therefore, we considered the IAA-induced changes to represent phenotypic plasticity, because the changes were always associated with population fitness and were reversible when the pressure was removed.

### IAA-induced morphological changes in *D. komarekii* is affected by competition against *Pectinodesmus pectinatus*

**Figure 9:**
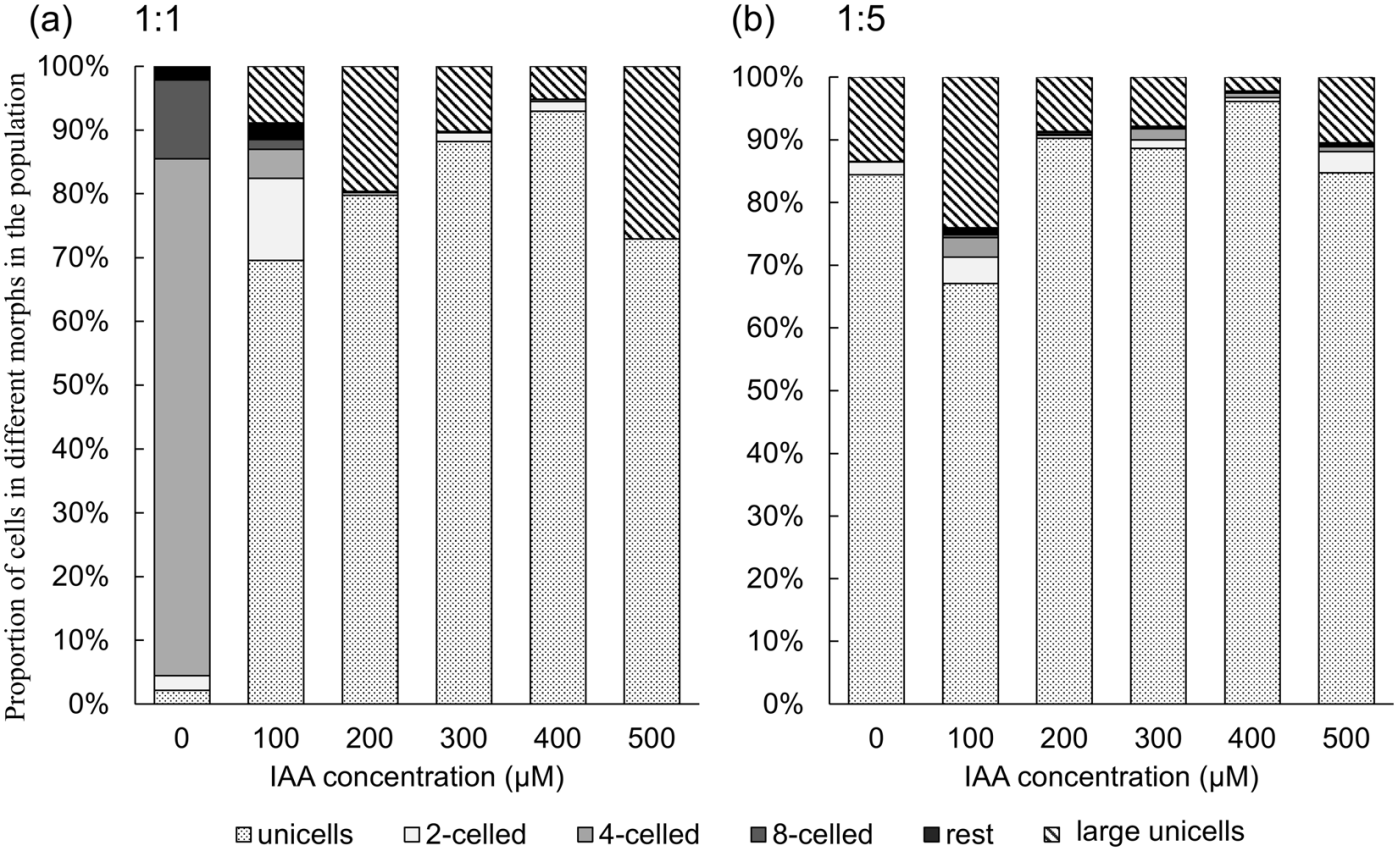
Proportions of unicells and 2-, 4-, and 8-celled coenobia in *D. komarekii* between control and IAA-treated groups in the presence of *P. pectinatus* with different initial densities. (**a**) Initial densities were 1:1 and (**b**) 1:5 at different concentrations of IAA. The “rest” group represents 3-, 5-, 6-, and 7-celled colonies. Data are presented as mean (n = 3) for each group, and morphotype percentages and cell types were based on 200 cell counts in each repeat.

**Figure 10:**
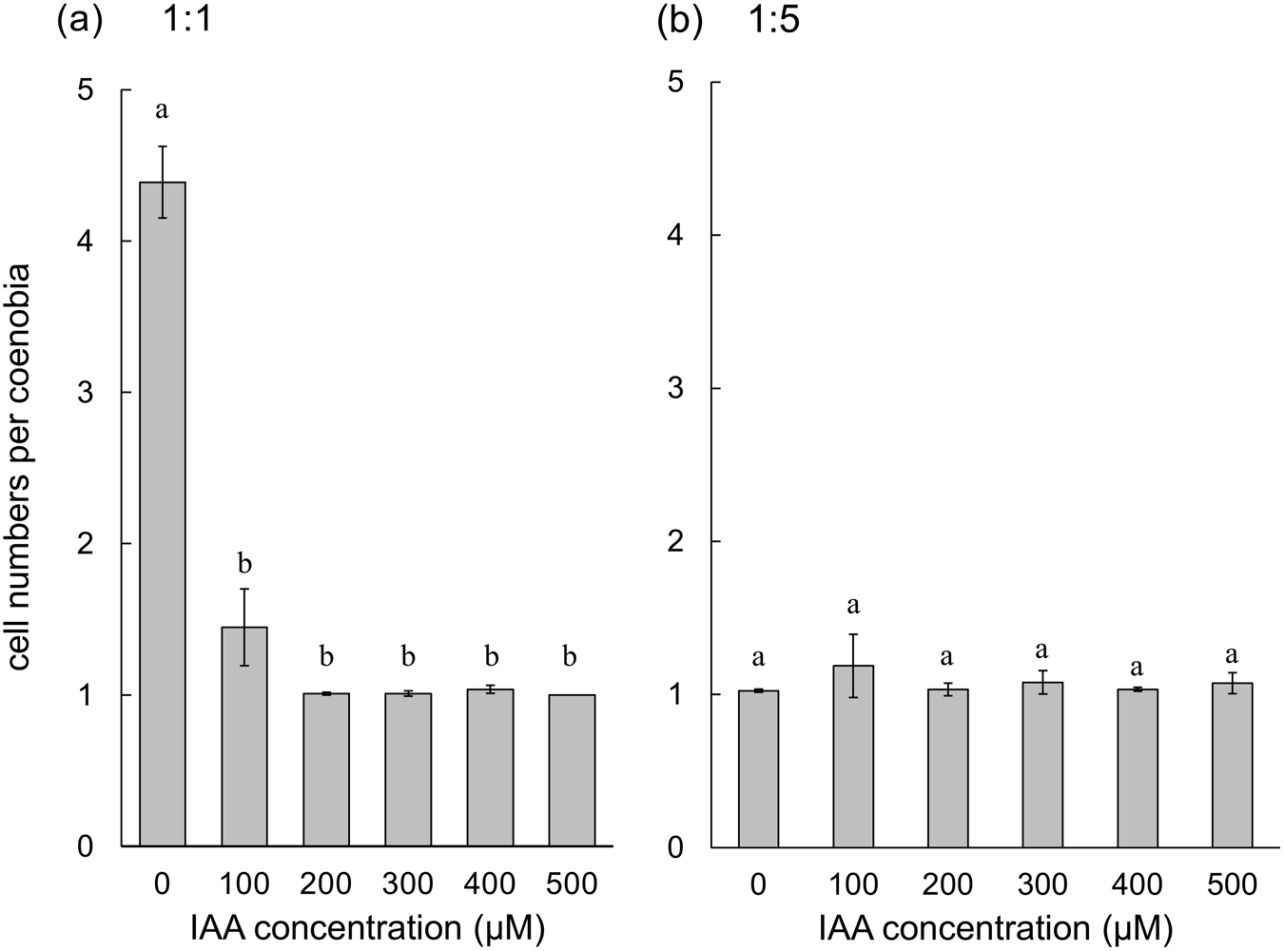
Mean number of cells per coenobium of *D. komarekii* between control and IAA-treated groups in the presence of *P. pectinatus* with different initial densities. (**a**) Initial densities were 1:1 and (**b**) 1:5 at different concentrations of IAA. Data are presented as mean ± standard error (n = 3) for each group. Morphotype percentages and cell types were based on 200 cell counts in each repeat. Means with the same letter are not significantly different from each other according to a one-way ANOVA with Bonferroni-Holm post-hoc test.

In the coculture experiment, the *D*. *komarekii* populations in the control groups were dominated by four-celled colonies as the cells in the monocultures (Fig. 9a). However, the morphological plasticity of the *D*. *komarekii* populations induced by IAA was drastically altered when *P*. *pectinatus* was added as a competitor to the *D*. *komarekii* cultures. The induced morphological changes in *D*. *komarekii* increased significantly in the presence of *P*. *pectinatus* under IAA treatment (compare Figs 2b and 9a). It means that IAA inhibited the colony formation, and the presence of competitor further enhanced this inhibition. The mean number of cells per particle of *D*. *komarekii* in the IAA-treated groups cocultured with 1-fold *P*. *pectinatus* reached its minimum at an IAA concentration of approximately 200 µM (Fig. 10a). In cocultures of *D*. *komarekii* and *P*. *pectinatus*, the large unicells were observed at an IAA concentration of 100 µM (Fig. 9a), but they only occurred in the monocultures of *D*. *komarekii* at IAA concentrations of more than 300 µM (Fig. 2b). Notably, in the cocultures with 5-fold *P*. *pectinatus*, the proportion of unicells was approximately 85%, even without IAA treatment (Fig. 9b). Additionally, the large unicells occurred in the cultures without IAA treatment. The mean number of cells per particle of *D*. *komarekii* in the IAA-treated groups cocultured with 5-fold *P*. *pectinatus* was not significantly different between the control groups and IAA treatment groups (Fig. 10b). Furthermore, in this competition experiment, we stained living *D*. *komarekii* and *P*. *pectinatus* cells with fluorescent dyes. The results showed that the *D*. *komarekii* cells and *P*. *pectinatus* cells in the control groups (without IAA treatment) did not produce, or only produced a small amount of, lipid droplets, but the large unicells of *D*. *komarekii* and *P*. *pectinatus* cells both accumulated a large amount of lipid droplets at an IAA concentration of 500 µM (Supplementary Figs 4 and 5).

## Discussion

The conenobial algae *Scenedesmus*, *Desmodesmus*, and *Pediastrum* are characterized by their remarkable phenotypic plasticity. Previous studies have demonstrated that the phenotypic plasticity of *Scenedesmus* is in response to grazing risk^11^. The *Daphnia* filtrate has been reported to stimulate the colony formation of *S. obliquus* from unicells to large colonies to avoid predators grazing on them^16,40^. The *Daphnia* filtrate was also indicated to induce defense colonies in some spiny *Desmodesmus* species^11^. Research has reported that the morphological changes of *Scenedesmus* were not only engendered by grazing pressure but also induced by heavy metals^12,41^ and indigo dye^42^. In our study, we revealed that exogenous IAA could induce morphological changes in *Desmodesmus*. We found that when the IAA concentration increased, the mean number of cells per particle of *Desmodesmus* decreased. We also discovered that specific large unicells were observable at a high IAA concentration. Microalgae absorb nutrients through the cellular membrane. Previous studies have reported that a reduction in colony size increases algal surface-to-volume ratios, and a high surface-to-volume ratio can improve light capture and nutrient uptake^43,44^. The advantages of unicells also including a slower sinking and it may indirectly influence the competitive ability of microalgae. The cell size influences the capacity of microalgae to be suspended in the euphotic zone^37^. The results from this study support that the organisms can overcome trade-offs to allocate limited resources to grow or to counteract stress^45^.

Biotic factors were determined to induce this phenotypic change, as also observed in *Pediastrum*. The *Pediastrum* population structure was different in the growth period compared with the stationary phase. In the early stage of culturing, the dominant shapes in populations were the eight-celled colonies whose cells were mainly small. The *Pediastrum* population structure changed over time. During the exponential growth phase, the relative number of single cells and the proportion of large cells within colonies increased, representing a bimodal distribution. When the algal cells reached the maximum density in the culture, a relative increase in single cells as well as in small cells in new colonies occurred. Notably, during the stationary period, this trend was reversed; fewer single cells and a larger cell size were observed^46^. This indicates that the nutrient supply could affect population structure, diminishing the proportion of eight-cell colonies.

IAA changed not only the morphology but also the physiology of *D. komarekii*. At a high IAA concentration, the growth of *D. komarekii* was suppressed, indicating that the environment was unfavorable to microalgae. When microalgae are under stressful conditions, they store their energy in lipid droplets, thus enabling them to survive under such conditions. Several factors such as nitrogen deficiency^47^, low temperature^48^, and high salinity^49^ have been demonstrated to induce an increase in the lipid content of microalgae, which assists microalgae in overcoming stressful conditions. In this study, we observed that the large unicells accumulated lipid droplets in their bodies, but other cell types did not produce lipid at a high IAA concentration. IAA treatment influenced not only the growth but also the physiological response of these cells. Despite the inadequacy of the cells’ photosynthetic efficiency, they were determined to use two other strategies to survive: (1) shrinking to facilitate nutrient uptake and light capture, and (2) producing large amounts of neutral lipids and altering metabolic pathways. According to the results, IAA might behave as a diffusible signal to facilitate interspecies communication. This phenomenon suggests the existence of a “quorum-sensing” mechanism that has evolved in microalgae to monitor the population density of competitors by sensing the accumulation of IAA excreted into the environment.

Notably, the proportion of infochemical-induced phenotypic changes in cultures was dependent on the relative abundance of competitors. Zhu *et al*.^16^ revealed that the grazer-induced morphological defense mechanism in *S*. *obliquus* was affected by its competition against another alga, *Microcystis aeruginosa*. In their study, they revealed that at a low intensity of competition, a large number of eight-celled colonies of *Scenedesmus* were formed, but at the expense of a decrease in competitive inhibition on the competitor *M*. *aeruginosa*. By contrast, the defensive colony formation of *S*. *obliquus* decreased at a high intensity of competition, facilitating the maintenance of a high competitive ability^16^. Therefore, *Scenedesmus* formed different morphologies in a trade-off between defense and competition. However, in our study, we found that the IAA-induced morphological features of *D. komarekii* were significantly altered when *Pectinodesmus pectinatus* was added as a competitor to the *D. komarekii* cultures. Here we found that IAA inhibited the colony formation, and the presence of competitor further enhanced this inhibition. In addition to the possible selection pressure by competitors, *P*. *pectinatus* may interfere with the regular response of *D*. *komarekii* by producing IAA. However, both *D*. *komarekii* and *P*. *pectinatus* did not demonstrate the ability to produce IAA after 7 days of culturing in our study (colorimetric Salkowski assay). It is possible that the competition for nutrients contributed to the morphological responses. However, additional studies are also necessary to determine what types of infochemicals are secreted from the algal competitor (*P*. *pectinatus*) to influence the sensitivity of the phenotypic plasticity of microalgae to IAA and also influence the morphology of microalgae, even without external IAA.

When many species are competing for the same finite resource, the theory of competitive exclusion (or Gause’s Law of competitive exclusion) suggests that two species cannot stably coexist, which limits biodiversity. However, this is not observed in nature. In aquatic biology, the paradox of the plankton describes a notable situation in which many species coexist on limited resources given the tendency for competition to exclude species. Over the past five decades, scientists have proposed that the paradox can be resolved by many specific factors that enhance the coexistence of multiple species^50^. However, most of the conditions are not always met, and many factors are not inherent to the offspring of species and are thus not subject to the mechanism of natural selection. Menden-Deuer and Rowlett introduced a game-theoretic model to support that the individual variability (such as phenotypic plasticity) in planktonic microorganisms is the key characteristic that ultimately enables coexistence and solves the paradox^51^. In this model, each individual only represents a small fraction of the clonal organisms, where the survival of each individual is associated with only a low risk. In this study, we employed IAA to act as a signal molecule in microorganisms to simulate an interspecific competitor. The morphological and physiological plasticity of *Desmodemus* in response to infochemicals altered the vertical distribution patterns of different cell types. Furthermore, we used another coenobial alga, *P. pectinatus*, to imitate algal–algal competition. We discovered that coexisting algal species (under *Desmodemus*: *Pectinodesmus* = 1:1 condition) exacerbated the simulated selection pressures from interspecific competition (IAA) on *Desmodemus*. Thus, the plasticity involving individual-level heterogeneity in behaviors and physiological characteristics is crucial for planktonic microorganisms to adapt to changing or novel conditions.

In summary, IAA induced phenotypic plasticity in *D*. *komarekii*, namely morphological and physiological changes. A low IAA concentration promoted the growth of *D*. *komarekii*, but a high IAA concentration exhibited an inhibitory effect. The mean cell number of coenobia and the chlorophyll *a*/*b* ratio decreased when the IAA concentration was increased. Large unicells appeared at a high IAA concentration and accumulated lipid. The presence of the competitor *P*. *pectinatus* promoted large unicell and unicell formation in *D*. *komarekii*. The proportion of unicells and large unicells in cultures was dependent on the relative abundance of competitors. At a high intensity of competition, a large number of unicells were formed and large unicells appeared early, which may have helped the microalgae to adapt to stressful conditions. *D*. *komarekii* exhibited different responses to different concentrations of IAA (as a simulated interspecific competitor) at different intensities of competition. These results demonstrate that IAA could be used as a diffusible signal to elicit interspecific communication among different organisms. Additionally, phenotypic plasticity assists microalgae in engaging in controllable phenotypic changes to reduce fitness costs by optimizing the cost–benefit ratios when responding to environmental challenges.

## Electronic supplementary material

Supplementary Infomation
