# Supplementary material for: Indole-3-acetic-acid-induced phenotypic plasticity in Desmodesmus algae
Source: Sci Rep. 2018 Jul 6;8:10270. doi: 10.1038/s41598-018-28627-z (PMC6035231; doi:10.1038/s41598-018-28627-z)
Supplement: Supplementary file 1 — Supplementary Infomation [file 41598_2018_28627_MOESM1_ESM.doc]

**Indole-3-acetic-acid-induced phenotypic plasticity in *Desmodesmus*** **algae**

Tan-Ya Chung, Chih-Yen Kuo, Wei-Jiun Lin, Wei-Lung Wang & Jui-Yu Chou*

Department of Biology, National Changhua University of Education, Changhua 500, Taiwan

*Corresponding author. Tel: +886-4-7232105 ext. 3428; Fax: +886-4-7211156; Email: jackyjau@cc.ncue.edu.tw

**Supplementary figures**

**
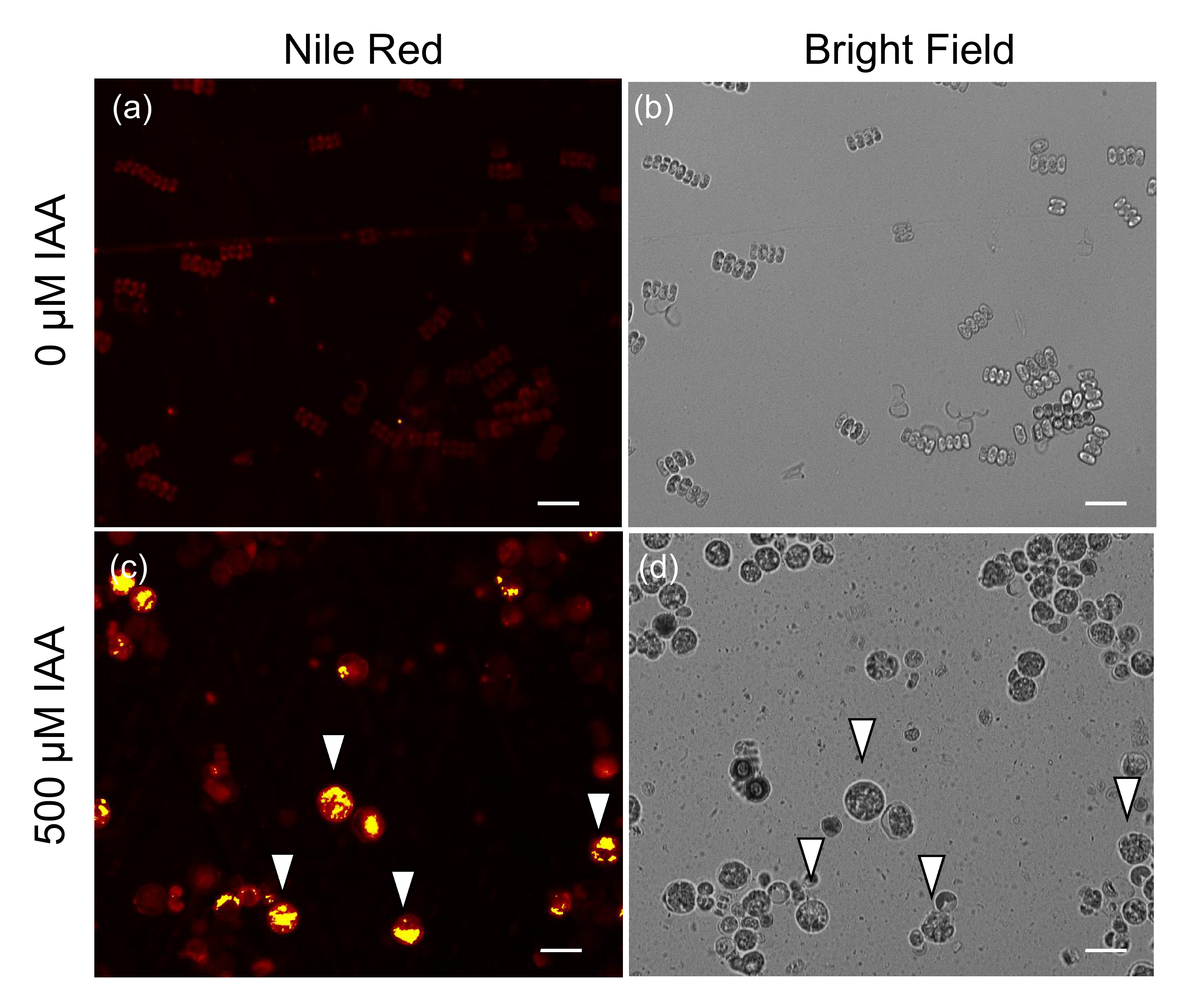
**

Supplementary Figure 1. *D. komarekii* were stained with Nile red. Arrowheads indicate the large unicell populations. (a, b) Control group: (a) lipids visualized by Nile red staining and (b) bright field. (c, d) Cultured under 500 µM IAA: (c) lipids visualized by Nile red staining and (d) bright field. The yellow fluorescence was from oil bodies and the red fluorescence was from chloroplasts. Bar: 20 μm.


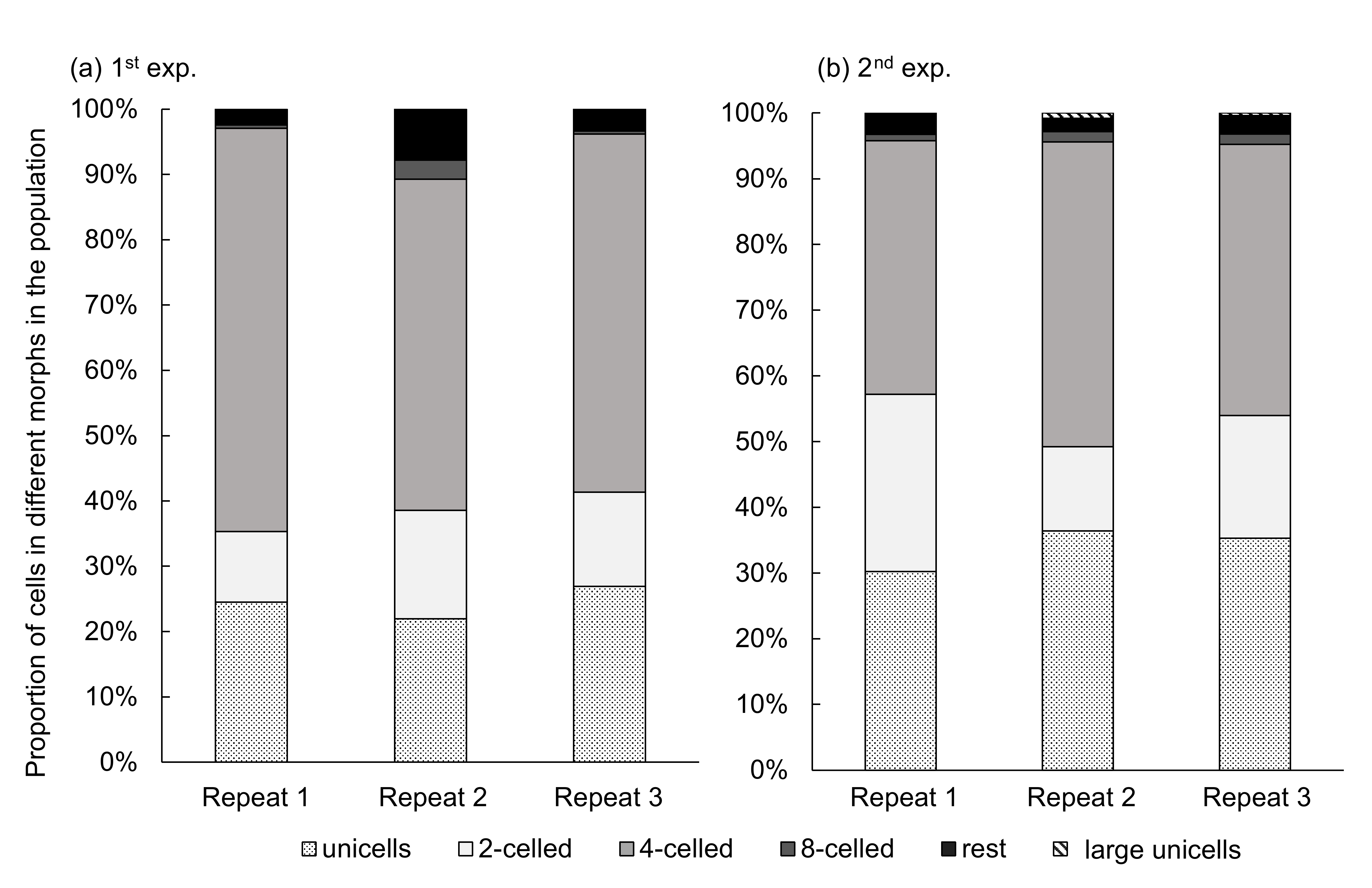


Supplementary Figure 2. Proportions of cells in different morphs in *D*. *komarekii* populations exposed to the high concentrations of IAA were transferred to fresh CA media, in which they gave rise to typical unicellular, two-celled, and four-celled dominant populations. The experiment was performed twice, and the two experiments yielded similar results. Additionally, for each experiment, three repeats were conducted. Proportions of unicells and 2-, 4-, and 8-celled coenobia of *D. komarekii* in the (a) first and (b) second experiments. The “rest” group represents 3-, 5-, 6-, and 7-celled colonies. Data are presented as mean (n = 3) for each group, and morphotype percentages and cell types were based on 200 cells counts in each repeat.


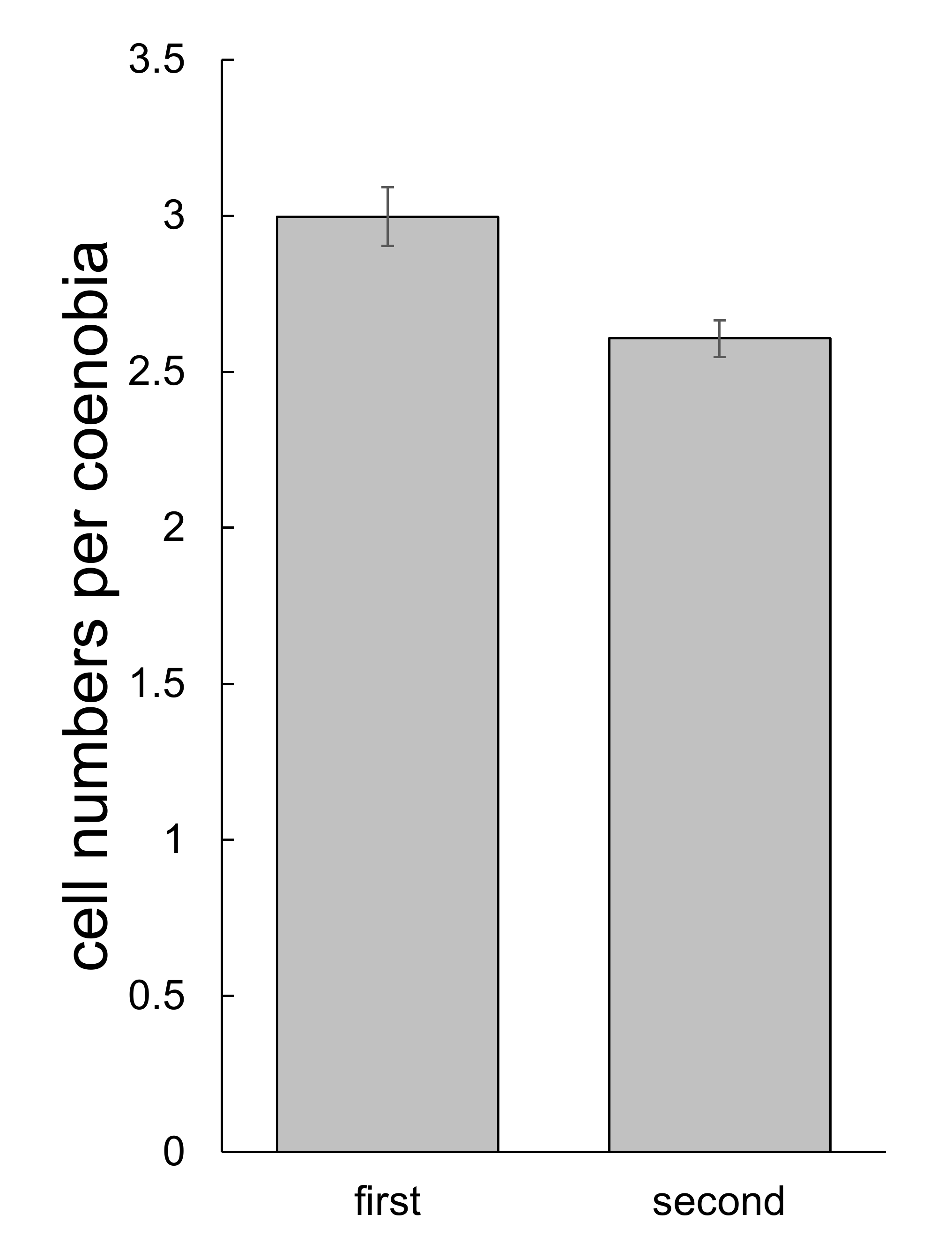


**Supplementary Figure 3. Cell numbers per coenobia in *D*. *komarekii* populations exposed to high concentrations of IAA were transferred to fresh CA media, in which they gave rise to typical unicellular, two-celled, and four-celled dominant populations. The experiment was performed two times, and the two experiments yielded similar results. Additionally, for each experiment, three repeats were conducted.** The mean number of cells per coenobia of *D. komarekii* in the (a) first and (b) second experiments. Data are presented as mean ± standard error (n = 3) for each group. Morphotypes percentages and cell types were based on 200 cell counts in each repeat.


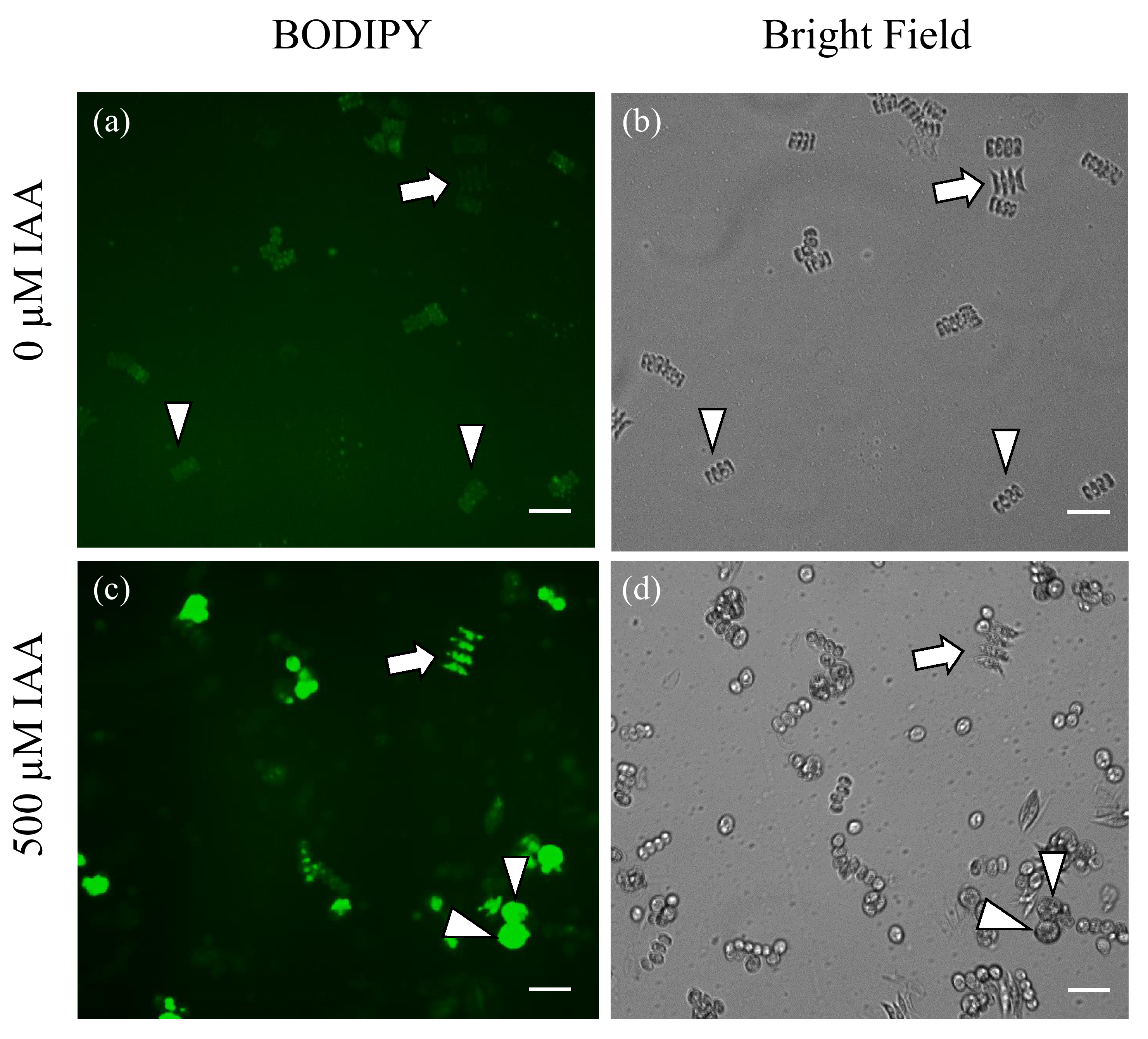


Supplementary Figure 4. *D. komarekii* and *P. pectinatus* were stained with BODIPY with the same density. Arrowheads indicate the *D*. *komarekii* populations, and the arrows indicate the *P. pectinatus* populations. (a, b) Control group: (a) lipids visualized by BODIPY staining and (b) bright field. (c, d) Cultured under 500 µM IAA: (c) lipids visualized by BODIPY staining and (d) bright field. Bar: 20 μm.


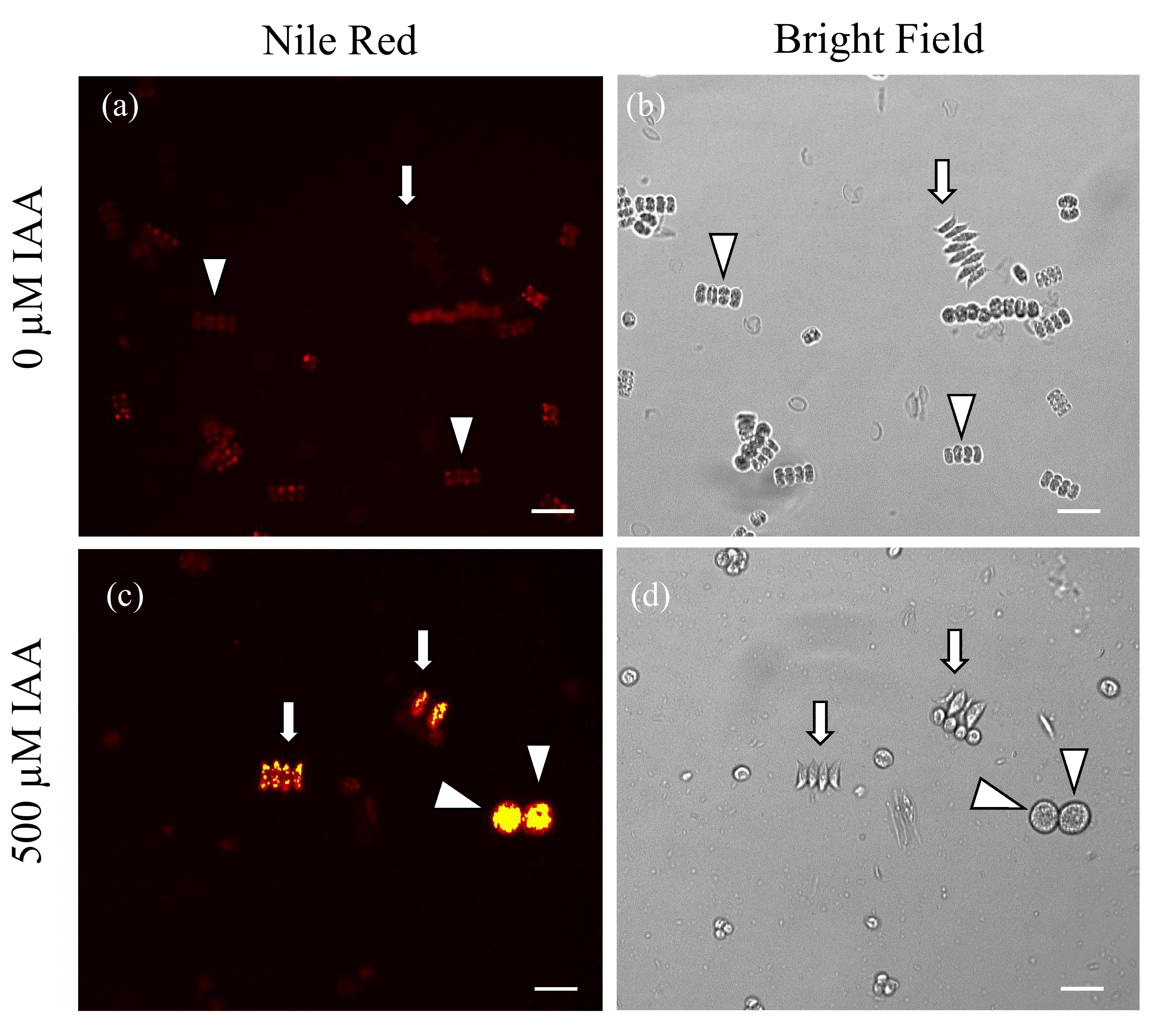


Supplementary Figure 5. *D. komarekii* and *P. pectinatus* were stained with Nile red with the same density. Arrowheads indicate the *D*. *komarekii* populations, and the arrows indicate the *P. pectinatus* populations. (a, b) Control group: (a) lipids visualized by Nile red staining and (b) bright field. (c, d) Cultured under 500 µM IAA: (c) lipids visualized by Nile red staining and (d) bright field. The yellow fluorescence was from oil bodies and the red fluorescence was from chloroplasts. Bar: 20 μm.
